# Direct Extracellular Electron Transfer of the *Geobacter sulfurreducens* Pili Relevant to Interaromatic Distances

**DOI:** 10.1155/2019/6151587

**Published:** 2019-11-11

**Authors:** Chuanjun Shu, Qiang Zhu, Ke Xiao, Yue Hou, Haibo Ma, Jing Ma, Xiao Sun

**Affiliations:** ^1^State Key Laboratory of Bioelectronics, School of Biological Science and Medical Engineering, Southeast University, Nanjing 210096, China; ^2^Department of Bioinformatics, School of Biomedical Engineering and Informatics, Nanjing Medical University, Nanjing 211166, China; ^3^Institute of Theoretical and Computational Chemistry, School of Chemistry and Chemical Engineering and Key Laboratory of Mesoscopic Chemistry of MOE, Nanjing University, Nanjing 210023, China

## Abstract

Microorganisms can transfer electrons directly to extracellular acceptors, during which organic compounds are oxidized to carbon dioxide. One of these microbes, Geobacter sulfurreducens, is well known for the “metallic-like” conductivity of its type IV pili. However, there is no consensus on what the mechanism for electron transfer along these conductive pili is. Based on the aromatic distances and orientations of our predicted models, the mechanism of electron transfer in the *Geobacter sulfurreducens* (GS) pili was explored by quantum chemical calculations with Marcus theory of electron transfer reactions. Three aromatic residues from the N-terminal *α*-helix of the GS pilin subunit are packed together, resulting in a continuous pi-pi interaction chain. The theoretical conductance (4.69 *μ*S/3.85 *μ*S) of the predicted models is very similar to that in the experiments reported recently (3.40 *μ*S). These findings offer a new concept that the GS pili belongs to a new class of proteins that can transport electrons through pi-pi interaction between aromatic residues and also provide a valuable tool for guiding further researches of these conductive pili, to investigate their roles in biogeochemical cycling, and potential applications in biomaterials, bioelectronics, and bioenergy.

## 1. Introduction

Direct Extracellular Electron Transfer (DEET) offers the possibility of novel, sustainable, cost-effective bioenergy strategies, and cleaning up environmental contaminants associated with traditional sources of energy [1]. These are attractive because the oxidation of the organic compound only releases fixed carbon back into the atmosphere. *Geobacter sulfurreducens* (GS), a microbe well known for its capability of DEET, can grow in subsurface groundwater contaminated via metallic oxides and bioremediate water polluted by uranium or hydrocarbons and similar pollutants associated with the mining and processing of nuclear fuel [2–5].

The process of DEET along the GS pili exhibits “metallic-like” conductivity similar to those of semiconductors [1, 6]. However, there was no explicit relationship between structure and electric conductivity at the molecular level of the GS pili, because of the difficulties in purifying pili crystals, i.e., the insolubility of subunits, the appendages on the surface of pilus, and the heterogeneity in the assembly of pilus [7, 8]. Therefore, the mechanism of DEET within producing bioenergy and restoring environmental pollutants for GS are not yet fully defined.

Because the c-type cytochromes are distributed on the surface of the GS pili, some studies attributed the electrical conductivity to the electron hopping/tunneling between these cytochromes [9–13]. Meanwhile, a previous study indicated that GS nanowires are assembled by micrometer-long polymerization of the hexaheme cytochrome OmcS, with hemes packed within ∼3.5–6 Å of each other [14]. However, direct imaging by atomic force microscopy revealed that the number of cytochromes on the surface of the GS pili is insufficient for electron transfer along the pili, and the cytochrome inhibition experiments also suggested that there is no correlation between cytochrome abundance and pili conductivity [11]. Hence, there is a huge controversy on the identification of DEET. Some studies focused on the pili itself [7, 15–17]. In the GS subunit, there are six aromatic amino acids (F1, F24, Y27, Y32, F51, and Y57). Furthermore, aromatic amino acids can transfer electrons among different redox centers [18]. Previous experiments of site-directed mutagenesis suggested that the GS pili transfer electrons along pili is at least partly attributed to aromatic amino acids [15, 16, 19–21]. Then, one kind of proposed mechanism of DEET for the GS pili is attributed to electron hopping between aromatic residues [22, 23]. However, it cannot adequately interpret the high conductivity (18.75 S·m^−1^) of the GS pili [16, 17, 22, 23]. Therefore, the role of aromatic residues in promotion of long-distance electron transport has not been fully established.

In the area of molecular crystalline organic semiconductors, charge-carrier mobilities can be described by any one of the three electron transport regimes, i.e., “band-like,” “hopping/tunneling,” and “pi-pi interaction” [24–26]. Meanwhile, the conductivities of robust pi-conjugated polymers may exceed 100 S cm^−1^ [27]. The high efficiency charge transport need for robust electronic transfer between subunits imposes rigorous requirement upon the intermolecular distances and orientations. Therefore, an understanding of the structural details of pili can provide new insights in their functions and strategies for extending these functions [28].

In this study, the relationship between molecular structure and biological function (electric conductivity) for the GS pili was explored by a combination of computational biology and computational chemistry. The energy-minimized conformations of the GS pili were constructed by methods of computational biology. The electrochemical basis for metallic-like conductive pili was interpreted from the perspective of computational chemistry. Structural and electrochemical parameters provide significant novel structural insights into the potential mechanism of the special conductivity of the GS pili. The pili conformations were generated by using homology modeling (Rosetta symmetric docking) and molecular dynamics simulations (MD simulations). This method optimizes side chain, backbone, and rigid-body degrees of freedom at the same time. The conductivities and conductance of pili were then calculated using the Marcus theory of electron transfer reactions based on aromatic distances and orientations in predicted pili conformations and compared with the biological experimental data [10, 29, 30]. According to conductivities and conductance between aromatic units within predicted pili models without extracellular redox mediators, the potential mechanism of DEET of the GS pili was firstly defined from the perspective of computational chemistry of organic semiconducting materials.

## 2. Results and Discussion

### 2.1. Structural Basis of Electron Transfer for the GS Pili

**Figure 1:**
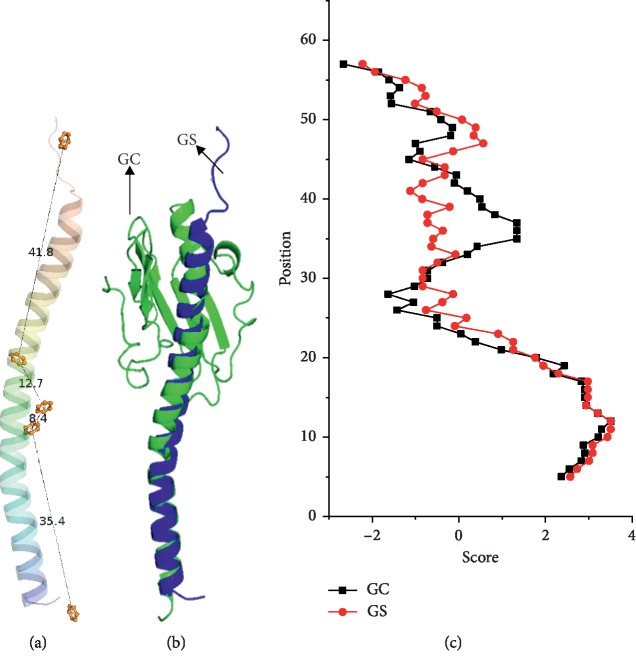
The structure and hydrophilic properties of GS and GC pili. (a) The spatial location of aromatic residues within GS subunit. The number and yellow stick represent interaromatic distance and aromatic ring, respectively. (b) GS pilin model is aligned with GC pilin. The blue and green cartoons represent GS and GC pilin, respectively. (c) The hydrophobicity plots of GS and GC pilin. The score is utilized to show hydrophobicity of residues. Red and black dots represent the residues of GS and GC pilin, respectively.

The GS pilin (PDB ID : 2M7G) was first refined in solution by MD simulation based on the steepest descent algorithm and the conjugate gradient algorithm (Amber 12) [31, 32], because its nuclear magnetic resonance conformation was derived from pilin subunits submerged in lipid micelles [33]. The distances between proximal aromatic ring centers were defined as interaromatic distances, and interaromatic distances for each pair of aromatic residues in the refined GS pilin were then measured (Figure 1(a)). These distances range from 8.7 Å to 41.8 Å, which suggest that aromatic residues in independent GS pilin could not form pi-pi interactions and thus could not construct an efficient electron transfer pathway. Therefore, possible aromatic contacts should be examined in the GS pili superstructure. The structures of the GS pili were constructed by assembling the refined GS pilin [34].

As depicted in Figure 1, the three-dimensional structure of the GS pilin shares remarkable structural similarity to the *Neisseria gonorrhoeae* (GC) pilin (Figure 1(b)), the subunit of another type IV pili [28]. The root mean square deviation (RMSD) between alpha-helical contents of the GS and the GC pilin was 2.55 Å. Furthermore, hydrophobicity plots of the GS and the GC pilin were very similar (Figure 1(c)). The high structural and hydrophilic conservation of the GS pilin with the GC pilin suggested that their assembly into pili structures would exhibit similar packing. Meanwhile, the intact structures of type IV pili that belong to right-handed helical symmetric proteins are similar [28, 35]. Therefore, helical symmetric parameters of the GS pili were deduced from the GC pilus of which the structure has been obtained by a combination of cryo-electron microscopy and x-ray crystallography [36].

**Figure 2:**
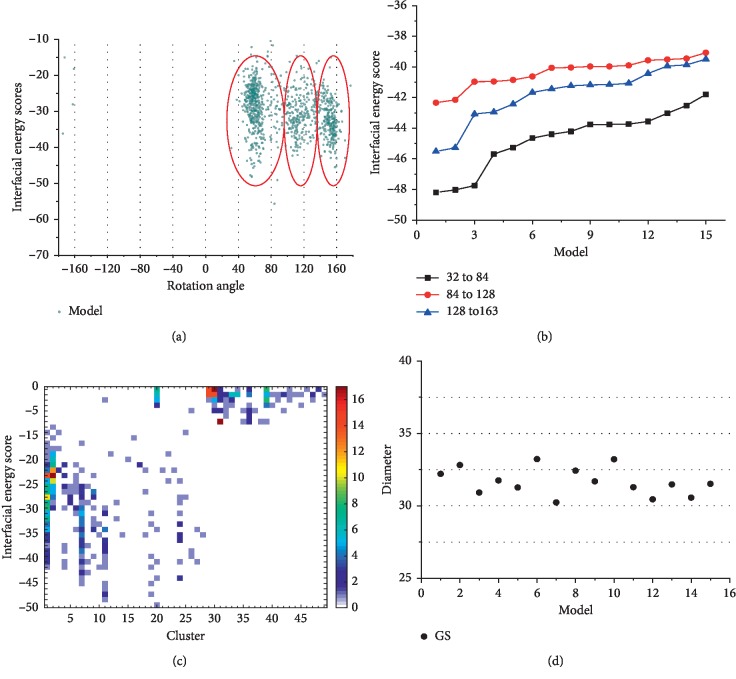
The structural details of GS pili. (a) The landscapes of interfacial energy score versus rotation angle for the GS pili. These red circles are utilized to highlight the converged troughs (rotation angles of 32° to 84°, 84° to 128°, and 128° to 163°). (b) The landscapes of the 15 lowest energy models in the biggest cluster for each of three converged troughs in the low-resolution phase. (c) The cluster details for high-resolution models of the GS pili. There are forty-nine clusters. The number of models is indicated with different colors. The cluster 1 is the biggest cluster. There are most models (16 models) in the energy score section that ranged from −24 to −25, compared to the number of models in other energy score sections. (d) The diameters of the 15 lowest energy models in the biggest cluster for the high-resolution GS pili. The unit of diameter is angstrom.

Ambiguous distance constraints were applied to the target pairs of amino acids (charged amino acids and aromatic amino acids) from adjacent pilins, to narrow down the sampling space [7, 34, 37]. A flat harmonic function was used to describe the distance constraints [7, 34]. The assembly procedure involved two stages: a low-resolution phase and a high-resolution one. In the high-resolution step, side chains were added, small perturbations were applied to the subunits, and a final annealing step was used to relax the full atomic models. Details of helical parameters and constraints are given in Supporting Information. In the low-resolution phase, 1000 low-resolution models were obtained. The rotation angles of these models could be clustered into three groups (rotation angles of 32° to 84°, 84° to 128°, and 128° to 163°) via k-means cluster analysis based on rotation angles [38] (Figure 2(a)). The cluster results were also obtained via DBSCAN (Density-Based Spatial Clustering of Applications with Noise) [39]. Then, the rotation angles could be clustered into three groups (rotation angles of 44° to 72°, 96° to 128°, and 129° to 163°) via DBSCAN (epsilon = 3; minPts = 10) based on rotation angles (Supporting Information [Supplementary-material supplementary-material-1]). Compared with results of k-means, these three extents were contained in the three extents, i.e., rotation angles of 32° to 84°, 84° to 128°, and 128° to 163°, that were obtained by k-means. Hence, the cluster results for k-means and DBSCAN are similar (Supporting Information [Supplementary-material supplementary-material-1]). For reducing false negatives, results of k-means were utilized for further analysis. Meanwhile, above 50% of low-resolution models have a rotation angle of 32° to 84° (Figure 2(a)). To reduce the range of sampling, 1000 low-resolution models were further obtained for each of the three convergent groups. We took the interfacial energy into account as it is an approximation to binding energy and depicted the stability of protein docking. The models that have a rotation angle of 32° to 84° had lower interfacial energy scores, compared to interfacial energy scores of models that have rotation angles of 84° to 128° and 128° to 163° (Figure 2(b)). These results suggested that the rotation angle of the native GS pili structure was most likely situated within the rotation angle of 32° to 84°. Therefore, the rotation angle of 32° to 84° was then utilized in high-resolution phase. The remaining helical symmetry parameters of initial structure for high-resolution phase were set as the same values in the model that has the lowest interfacial energy in the biggest cluster of the low-resolution phase. In the high-resolution phase, 1000 full atomic models were then obtained. We used a combination of clustering and energy score to evaluate our models, because native structure is situated in a wide basin of low-energy conformations (the biggest cluster), for keeping the robustness and efficiency of structure [34, 37]. The full atomic models were removed when they were unstable (energy score exceed zero). After a clustering process based on structural similarity (“cluster” module in Rosetta, cutoff = 1.75 Å), the stable full atomic models were divided into 49 clusters (Figure 2(c)), and there were 118 models in the biggest cluster (cluster 1 in Figure 2(c)). The diameters of the 15 lowest energy models in the biggest cluster were measured which was around 32 Å (Figure 2(d)), consistent with the previous observation value (30 to 40 Å) [1, 16]. The fluctuations of the observed diameters are caused by the heterogeneous surface of pilus.

**Figure 3:**
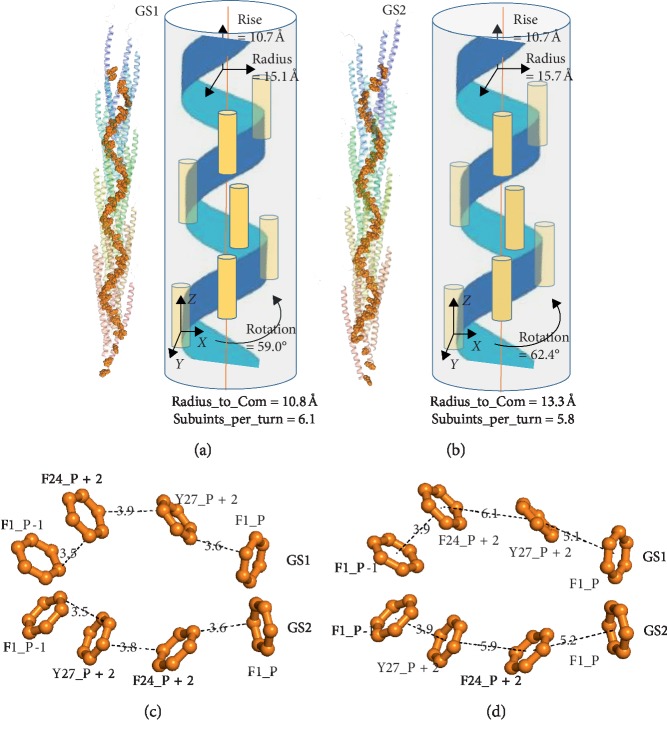
The spatial location of aromatic residues within GS pili. The helical symmetry parameters, i.e., rotation angle, radius, subunits_per_turn, rise, and radius_to_com, of (a) GS1 and (b) GS2 pili. Orange spheres and yellow columns represent aromatic residues and subunits, respectively. The proximal carbon-carbon distances and interaromatic distances are shown in picture (c) and (d), respectively. The unit of distance is angstrom. The smallest symmetric aromatic units in GS1 and GS2 pili are shown in picture (c) and (d). F1_P-1 and F1_P represent the start and end nodes of a smallest symmetric aromatic unit. *P* represents the first subunit of the second turn in pili models, which is set as 7.

Two models (GS1 and GS2) in the biggest cluster were selected for calculation of the conductivity (Figures 3(a) and 3(b)). Native models tend to converge at the point with the lowest interface energy score. In addition, most models in the largest cluster were near the lowest energy model, that is, the native model. Hence, GS1 was the lowest energy model of the biggest cluster, and GS2 was the lowest energy model from the energy concentration section (energy score of −24 to −25) in the biggest cluster. From the spatial arrangement of aromatic residues, there was a remarkable tightly packed aromatic chain in each of two models. A shortest pathway algorithm (*Dijkstra* algorithm) was utilized to investigate the potential electron transfer pathways in the GS structures [40]. In the two models, pi-orbitals of three aromatic residues, i.e., F1, F24, and Y27, from different subunits probably overlapped and were expected to provide a potential helical symmetric electron transfer pathway (Figures 3(a) and 3(b)). As shown in Figures 3(c) and 3(d), the smallest symmetric unit of helical electron transfer pathway consisted of four aromatic residues in both models. Meanwhile, the distances between proximal carbon atoms in neighbouring aromatic rings were defined as proximal carbon-carbon distances, and two types of distances, i.e., proximal carbon-carbon distances and interaromatic distance, were then measured by PyMOL package (Figures 3(c) and 3(d)) [41]. The proximal carbon-carbon distances in both GS1 and GS2 indicated that aromatic residues in the GS pili are packed in 3.5 to 3.9 Å, very close to the previous experiment results (3 to 4 Å) [42]. The three interaromatic distances, i.e., *d*_1_ (F1, F24), *d*_2_ (F24, Y27), and *d*_3_ (Y27, F1), were 3.9, 6.1, and 5.1 Å for GS1 pilus, and 5.2, 5.9, and 3.9 Å for GS2 pilus, respectively. Compared to previous models [7, 10, 21, 42], models in this manuscript have a closer aromatic residues chain and a more reasonable geometric configuration between aromatic residues. This result could be owing to three main aspects. Firstly, detailed structural and assembly models of the GS pili were obtained from monomeric data and sparse constraints [34, 37]. Type IV pili models that were predicted by this approach are closer to natural structure than other approaches [8]. Secondly, models in this manuscript were also refined by molecular dynamic simulation. Previous study indicated that molecular dynamic simulation could be utilized to improve the geometry and structural statistics of protein [21, 32]. Thirdly, a previous study has shown that the genetic substitution of an alanine for each of the three tyrosine (+27, +32, and +57) and two of the phenylalanine (+24 and +51) in PilA resulted in an assembled pilus with poor conductivity [15]. Genetic substitution of an alanine for the phenylalanine (+1) in PilA, the pilus subunit, resulted in assembled pili likely with poor conductivity, further indicating the importance of F1 aromatic amino acids in electron transport [43]. These results suggest that these genetic substitutions resulted in different GS pilus conformations, which probably resulted in various electron transfer rates of G. sulfurreducens pilus. Hence, the interaromatic distances were then utilized to quantitative evaluate charge transport properties of the GS pili.

### 2.2. The Charge Transport Properties of the GS Pili

A quantitative evaluation of conductivities was accomplished through the quantum mechanical electronic structure calculation with first principles density functional theory (DFT) by using Gaussian 09 package [44]. The molecular details, i.e., the spatial arrangement of the aromatic residues, interaromatic distances, and helical symmetry parameters, of the two pili models were incorporated into our calculation of the electron structures. The technical details of the computations of conductivities are described in Supporting Information. Here, the primary methods and conclusions are highlighted. Firstly, the reorganization energies of passivated aromatic residues in their corresponding spatial position were computed [45]. The electronic couplings between the pi-conjugated aromatic residues were calculated with DFT at the level of CAM-B3LYP/6-31G [46–48]. Electron transfer rates have a close relationship with the reaction transfer energies (Δ*G*°) that are greatly affected by environment [49–51]. This uncertainty disappears because we focus here on the upper limit of conductivities of the GS pili. Therefore, the upper limit of conductivity can be calculated by the prefactor of the Marcus formula [52]:

(1) $$\begin{array}{c} \;w\leq \;t^{2}\sqrt{\frac{\pi }{{ћ}^{2}\lambda kT}}, \end{array}$$

where *t* represents the electronic coupling and *λ* represents the charge transfer reorganization energy. The values of *λ* are about 0.37 eV for all the aromatic residues in electron transfer pathway.

The electronic transport of the GS pili was determined by the lowest transfer rates along electron transfer pathway. Since the GS pili belonged to a symmetric helical protein [28], the smallest symmetric unit of helical electron transfer pathway consisted of four aromatic residues in the GS pili models (Figure 3). Hence, there were three types of distance between aromatic residues in the GS pili (Figure 3). The largest distance restricted the electron transfer rates along the GS pili and resulted in the lowest transfer rates for charge mobility. Hence, the lowest transfer rate was employed to calculate charge mobility. As a result, for both GS1 and GS2 models, the calculated electron (*t*_e_) and hole (*t*_h_) transfer integrals exceed 2.93 × 10^−2^ eV and 1.46 × 10^−2^ eV, respectively; the computed electron and hole diffusion coefficients exceed 2.28 × 10^−6^ m^2^·s^−1^ and 0.57 × 10^−6^ m^2^·s^−1^, respectively; the estimated electron and hole charge motilities exceed 8.86 × 10^−5^ m^2^·v^−1^·s^−1^ and 2.19 × 10^−5^ m^2^·v^−1^·s^−1^, respectively (Table 1). Meanwhile, the computed carrier concentrations (*ρ*) for each of two models were about 4.00 × 10^26^ m^−3^. Under these conditions, the conductivities (*σ*) for each of two models were calculated by utilizing the relationship between conductivity, carrier concentration, and mobility (*μ*), i.e., *σ*=|*e*|*ρ*(*μ*_e_+*μ*_h_). The *σ* values of pili were 8601.51 S·m^−1^ and 7073.26 S·m^−1^ for GS1 and GS2, respectively (Table 1). These calculated values were far above the measured value that is obtained in previous experiment study, i.e., 18.75 S·m^−1^ (Table 1) [17, 22, 30]. Recently, the approach for measuring pili conductance was modified by Lovely group [29]. In this method, conductance was measured by the four-point probe method for eliminating the contact resistance between the electrodes and the GS pili specimens [29]. Our calculated theoretical conductance (4.69 *μ*S and 3.85 *μ*S) was very close to that of the recent experimental result (3.40 *μ*S) (Table 1) [29]. The factors that can be utilized to explain the difference between theoretical analysis and published experimental results include five main aspects: (i) the upper limit of transfer rates was computed without considering the local electrostatic environment around aromatic residues; (ii) these calculations were based on the smallest symmetry units without considering energy consumption during the electron transfer process in the pili; (iii) it neglected the influence of extracellular cytochromes [13]; (iv) it did not consider the perturbations by virtue of ion conductivity [54]; and (v) there were differences in pili aromatic spatial location between real protein conformations and the structures for calculations here.

**Figure 4:**
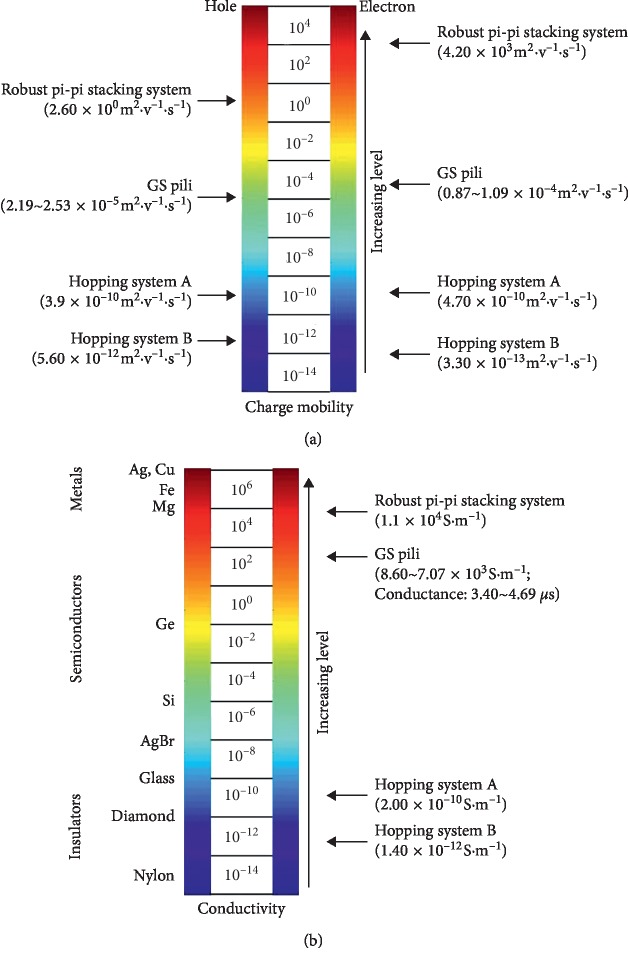
The charge mobilities and conductivities for different systems. Different colors are utilized to distinguish different systems. (a) The hole and electron charge mobility for each of the hopping system A, the hopping system B , the GS pili, and the robust pi-pi stacking system (perylene bisimide dyes) is 3.90 × 10^−10^ m^2^·v^−1^·s^−1^, 5.60 × 10^−12^ m^2^·v^−1^·s^−1^, 2.19 × 10^−5^ m^2^·v^−1^·s^−1^, and 2.60 × 10° m^2^·v^−1^·s^−1^, and 4.70 × 10^−10^ m^2^·v^−1^·s^−1^, 3.30 × 10^−13^ m^2^·v^−1^·s^−1^, 1.09 × 10^−4^ m^2^·v^−1^·s^−1^, and 4.20 × 10^3^ m^2^·v^−1^·s^−1^, respectively. (b) The conductivity of the hopping system A the hopping system B and the GS pili is around 10^−10^ S·m^−1^, 10^−12^ S·m^−1^, and 10^3^ S·m^−1^, respectively. The conductivity (1.10 × 10^4^ S·m^−1^) of the robust pi-pi stacking system (perylene bisimide dyes) belong to metallic-like materials.

A previous study suggested that the electron transfer is attributed to hopping when the hole and electron charge mobility of biological filament ranged from 5.60 × 10^−12^ m^2^·v^−1^·s^−1^ to 390.00 × 10^−12^ m^2^·v^−1^·s^−1^ and 3.30 × 10^−13^ m^2^·v^−1^·s^−1^ to 4700.00 × 10^−13^ m^2^·v^−1^·s^−1^, respectively (Figure 4(a)) [23]. Meanwhile, the charge mobility of robust pi-pi stacking system (perylene bisimide dyes) can reach 4.20 × 10^3^ m^2^·v^−1^·s^−1^ [26]. In this study, the hole and electron charge mobility of the GS pili ranged from 2.19 × 10^−5^ m^2^·v^−1^·s^−1^ to 2.53 × 10^−5^ m^2^·v^−1^·s^−1^ and 8.86 × 10^−5^ m^2^·v^−1^·s^−1^ to 10.90 × 10^−5^ m^2^·v^−1^·s^−1^, respectively (Table 1 and Figure 4(a)). These results suggested that the charge mobility of the GS pili fell in between the hopping system and the robust pi-pi stacking system (Figure 4(a)).

The calculated conductivities of the GS pili (ranged from 7.07 × 10^3^ S·m^−1^ to 8.60 × 10^3^ S·m^−1^) indicated that this pili belong to biological semiconducting materials (Table 1 and Figure 4(b)). Aromatic amino acids, capable of absorbing energy which excites electron to excited state, could function as intermediate acceptors or donors in electron transfer [55]. Attractive interactions (pi-pi interactions) between aromatic residues are one of the principal noncovalent forces governing energy flow in biological structure. Meanwhile, in a polymeric pilus structure, GS subunits assemble tightly together and aromatic residues are closely packed, resulting in pi-pi interactions. Furthermore, perylene bisimide dyes have the robust pi-pi stacking system, resulting in semiconducting conductivity (1.10 × 10^4^ S·m^−1^) [26]. Hence, both perylene bisimide dyes and the GS pili have semiconducting conductivity (10^−8^ to 10^4^ S·m^−1^) [1, 17, 26, 56]. However, the conductivity of hopping system ranged from 1.40 × 10^−12^ S·m^−1^ to 200.00 × 10^−12^ S·m^−1^ [23]. Therefore, according to spatial location of aromatic residues and calculated conductivities, we can speculate that the GS pili invoke the pi-pi interaction between aromatic residues for charge transport.

The GS pilin, without c-terminal globular domain, probably evolved from the conventional type IV pilin due to the pressure of extracellular electron transfer for bacteria that do not have direct contact with external electron acceptors [10]. The predicted pili models in this study indicated that the truncated pilins assemble tightly together, resulting in a continuous pi-pi interaction chain. Although proteins belong to typically insulators, the GS pili have metallic-like conductivity [3, 22]. The mechanism of direct extracellular electron transfer of the GS pili is interesting because these microbial “nanowires” play critical roles in biogeochemical cycling and applications in biomaterials, bioelectronics, bioremediation, and bioenergy. The calculated conductance described here further demonstrated that it is feasible for the GS pilins to pack into a highly stable conformation where a closely packed chain of aromatic residues facilitates electron transfer along the pilus via pi-pi interactions, resulting in the semiconducting conductivity.

## 3. Conclusions

In this study, energy-minimized structures of the GS pili were constructed by a combination of MD simulations and homology modeling. Three aromatic residues from N-terminal *a*-helix of the GS pilin were distributed in a potential conductive geometry, i.e., pi-pi interaction chain. According to the special aromatic distances and orientations within the GS pili, results of quantum chemistry calculation suggested that the metallic-like conductivity of the GS pili is attributed to a robust pi-pi interaction chain. The studies reported here offer significant electronic structural insights into the mechanism of extracellular electron transfer of the conductive pili and provide a novel concept that the GS pili are a new kind of electronically functional proteins, the conductivity of which is attributed to pi-pi interactions among aromatic residues. Furthermore, the studies reported here point out a potential relationship between protein molecular structure and electronic charge transport regime and provide new insight on the strategies of synthesizing high electrically conductive, nontoxic bioelectronic materials based on the correlation between protein structure and electronic charge transport.

## 4. Materials and Methods

### 4.1. Optimizing GS Pilin by MD Simulation

The structure of GS (*Geobacter sulfurreducens*) pilin was downloaded from the RCSB protein data bank (RCSB PDB) [57]. GS pilin was then subjected to molecular dynamics simulation using Amber 12 software (TRII-Biotech, Shanghai, China) with a lipid 11 force field, since the NMR-derived pilin structure was derived from pilin subunits immersed in lipid micelles [31]. Topology files of pilin were created using Leap module in Amber 12. The POPC membrane was added by utilizing CHARMM-GUI web server [58]. Two Na^+^ were added to maintain the neutrality of the simulated system. The pilin was then inserted in a regular tetrahedron water box of the TIP3P with a 10 Å distance around the protein. Finally, all the missing hydrogen atoms of pilin were added by the Leap module of the Amber 12.

Energy minimization, heating, and equilibration protocol were performed by the PMEMD (particle mesh Ewald molecular dynamics) module of Amber 12. The energy minimization consisted of two phases. Namely, the system were minimized when backbone atoms of the protein were constrained by a harmonic constraint potential with 100 kcal·mol^−1^·Å^−2^. The whole system was then minimized without any restraint. Each minimization phase was implemented by the steepest descent algorithm (50,000 steps) followed by a conjugate gradient algorithm (20,000 steps) where the nonbonded interaction cutoff was set to be 8 Å. After minimization, the system was gradually heated from 0 to 300 K using a Langevin thermostat with a coupling coefficient of 2 ps^−1^ in 500 ps molecular simulation (a canonical ensemble NVT). Next, systems were equilibrated for 0.5 ns in the isothermal isobaric ensemble (NPT). In the heating and equilibration phases, solute molecules were restrained by applying a constant harmonic force of 50 kcal·mol^−1^·Å^−2^ and 5 kcal·mol^−1^·Å^−2^, respectively. According to a previous study, the systems were then subsequently subjected to 125 ns MD (molecular dynamic) simulation in the NPT ensemble at 300K with a time step of 2 fs. In the MD simulation phase, 8 Å cutoff, SHAKE algorithm, particle mesh Ewald (PME), periodic boundary conditions, Langevin piston algorithm, and Berendsen barostat were used to treat the nonbonding interactions, perform constraint covalent bonds involving hydrogen atoms, treat long range interactions, and avoid edge effects in calculations, temperature, and pressure control. The coordinates were saved every 1 ps and the trajectory files were analyzed every 1 ps using the *Cpptraj* module implemented in the Amber 12 software.

### 4.2. Symmetric Docking with Sparse Constrains

To construct GS pilus superstructure, we applied the knowledge that type IV pili have similar intact structures with a right-hand helical symmetry along their assemblies. Subsequently, Rosetta was applied to construct pilus structure by assembling the refined subunits with symmetric docking and sparse constraints [57, 59]. To obtain appropriate structures, the assembly process involved two steps: low- and high-resolution phases. The low-resolution phase was performed to search for potential pilus structures from a wide sampling space. In the high-resolution phase, side chains were added, and a small initial perturbation and a fast simulated annealing step were implemented to produce full atomic models with structural details.

In two phases, helical symmetry was applied by defining a symmetrical conformation space through six degrees of freedom (DOFs) of the rigid-body: the distance between the axis and the center of mass (COM) of the subunits; the rotation around the axis; the translation along the axis; and three orientation dimensions of subunits (*x*, *y*, and *z*). In symmetric docking module of Rosetta, only one refined pilin was accounted for in the calculation, and all other subunits were translated from the refined pilin through the six DOFs. The symmetry information of the helix could be inferred from an experimentally determined homologous structure, i.e., the GC (*Neisseria gonorrhoeae*) pilus (PDB ID : 2HIL) [28, 36].

Proper distance constraints and ambiguous constraints between pairs of residues from adjacent subunits were utilized to narrow down the sampling space. Distance information was obtained from constrained pairs of residues (e.g., F1/E5 of T4P, V9/L16 of GC pilin) from known structural details and experimental results such as salt bridge charge reversal experiments, cysteine crosslinking, and hydrogen/deuterium exchange mass spectrometry [7]. Ambiguous contacts between two residues were also applied because of the arrangement of the subunits in GS pili was unknown. These ambiguous contacts were delineated as an enumeration of all combinations of the residue pairs, which were from two different subunits, C_1_, C_2_,…, C_*n*_. From min (C_1_, C_2_,…, C_*n*_), the ambiguous constraint was set on pairs that had a minimum from all the energy scores. Here, the total number of subunits was fixed to be 21 (chain names are denoted as consecutive letters: A, B, C, D,…, U). Owing to symmetry, ambiguous contact constraints were only added to the subunit in the middle and the upper ten subunits. To use constraint information during the assembly processes, the distance constrains between pairs of atoms were characterized as energy penalty functions for Rosetta. When the Euclidean distance between two atoms was either too small or too large, the function was performed to ensure that models were correctly penalized. A flat harmonic function was applied to fit distance constraints:

(2) $$\begin{array}{c} f\left(d\right)=\left\{\begin{array}{cc} 0, & \left|d-x_{0}\right|\leq t,\\ {\left(\frac{d-x_{0}-t}{s\;d}\right)}^{2}, & d>x_{0}-t,\\ {\left(\frac{d-x_{0}-t}{s\;d}\right)}^{2}, & d<x_{0}-t, \end{array}\right. \end{array}$$

where *x*_0_ is an approximation of distance between a pair of atoms, which represents the center of constrains; *sd* denotes standard deviations of Euclidean Distance (*d*) between two atoms; and tolerance (*t*) means the acceptable bound of constraints. These three variables were set to be *x*_0_ = 10, *sd* = 0.5, tolerance = 5, and the constraints were added to the C*α* atoms of each residue in the low-resolution phase. In the high-resolution phase, these three variables were set to be 4, 2, and 0.5, respectively, and the constraints were enforced on N-O atom pairs in salt bridges. These sparse constraints were applied with Rosetta Constraint Files.

Before the symmetric docking, the subunit was aligned along the pilus axis by PyMOL to define the initial position and facilitate the search process [41]. Symmetric docking of subunits with sparse constraints was implemented in Rosetta to obtain 1000 models of full atomic pilus models. We used a combination of a clustering process (Cut off = 1.75 Å) and energy scores of three consecutive subunits (chain names: E, F, and G), to evaluate the models obtained from symmetric docking. The “cluster” application in Rosetta was performed according to structure similarity. The algorithm of cluster definition was based on an old Bradley program (silent_cluster_c). For the low- and high-resolution models, both the total energy and interface energy of the subunits were applied. We considered interface energy in the high-resolution models because it can approximate binding energy and reflects the stability of pilin assembly.

### 4.3. Quantum Chemical Calculations

To explore the electron transfer mechanism, the charge transfer rates among aromatic residues were calculated using the Marcus formula. There are three key parameters need to be extracted from quantum chemistry calculations, namely, transfer integral, reorganization energy, and change in free energy. In this study, only the upper limit of transfer rates of pili was estimated, resulting in the Δ*G*° (change in free energy) disappears. Density functional theory (DFT) methodology was applied throughout this study for quantum chemistry calculation. All calculations are carried out using the Gaussian 09 program with CAM-B3LYP functional and 6-31G basis set [23, 44]. The transfer integrals are calculated by an energy-gap based scheme [60]:

(3) $$\begin{array}{c} t_{\text{hole}}=\frac{E_{\text{HOMO}}^{\text{dimer}}-E_{\text{HOMO}-1}^{\text{dimer}}}{2},\\ t_{\text{elec}}=\frac{E_{\text{LUMO}+1}^{\text{dimer}}-E_{\text{LUMO}}^{\text{dimer}}}{2}. \end{array}$$

The presence of excess charge on an organic molecule/unit will alter its geometry. The energy change due to such structural reorganization will act as a barrier for charge transport. Therefore, the structural reorganization energy is an important indicator for evaluating the charge-carrier mobilities. The reorganization energy is defined as follows [61]:

(4) $$\begin{array}{c} \lambda =\lambda^{D}+\;\lambda^{A}=\left(E_{cn}^{D}-E_{nn}^{D}\right)+\left(E_{nc}^{A}-E_{cc}^{A}\right), \end{array}$$

where *E*_*cn*_^*D*^ refers to the energy of the neutral donor residue in the optimized geometry of its charged state (the first subscript means the geometry and the second means the charge state).

The charge diffusion coefficient of the one-dimensional pilin structures is computed as follows [62]:

(5) $$\begin{array}{c} D=W_{0}Z_{0}^{2}, \end{array}$$

where *W*_0_ represents the effective rate and the *Z*_0_ represents the height of a unit periodic cell.

The charge mobility is then computed by the Einstein relation [63]:

(6) $$\begin{array}{c} \mu =\frac{e\;D}{kT}. \end{array}$$

To obtain the conductivity of the GS pili, the charge-carrier concentrations of aromatic amino acids were estimated. As an upper bound, the number density of the aromatic residues was taken as the density of free holes and electrons in the pili. Taking the radius of the aromatic packing to be *r*_0_ = 13 Å, the number density of aromatic residues is given by

(7) $$\begin{array}{c} \rho =\frac{4}{лr_{0}^{2}\times z_{0}}. \end{array}$$

The conductivity is given by [22]

(8) $$\begin{array}{c} \sigma =\left|e\right|\rho \left(\mu_{h}+\mu_{e}\right), \end{array}$$

where *e* is the charge of an electron and *μ*_*h*_ and *μ*_*e*_ are the mobilities of holes and electrons, respectively.

Finally, the conductance *G* is calculated by [29]

(9) $$\begin{array}{c} G=\sigma \frac{S}{l}, \end{array}$$

where *l* is the length of the conductor (interaromatic distance) and *S* is the cross-sectional area of the conductor measures in square meters (the area of the aromatic ring).

## Figures and Tables

**Table 1:** Charge transport properties of GS1 and GS2 pilus models and biological experiment values of GS pilus.

Charge carrier	GS1		GS		GS
	Hole	Electron	Hole	Electron	Pili
Transfer integral (eV)	1.56 × 10^−2^	3.25 × 10^−2^	1.46 × 10^−2^	2.93 × 10^−2^	N.A.
Diffusion coefficient (m^2^·s^−1^)	6.50 × 10^−7^	2.81 × 10^−6^	5.65 × 10^−7^	2.28 × 10^−6^	N.A.
Charge mobility (m^2^·v^−1^·s^−1^)	2.53 × 10^−5^	1.09 × 10^−4^	2.19 × 10^−5^	8.86 × 10^−5^	N.A.
Conductivity (S·m^−1^)	86.02 × 10^2^		70.73 × 10^2^		18.75^a^
Conductance (*μ*S)	4.69		3.85		3.40^b^

^a^The biological experimental conductivity of individual GS pili filament preparation obtained in biological experiment [16, 17], where the presence of extracellular in the preparation is under debate [6, 53]. ^b^The biological experimental conductance of GS pili is measured by a modified method [29].

## Data Availability

The original data used to support the findings of this study are available from the corresponding author upon request.

## Supplementary Materials

Supplementary Materials The supplementary material file consists of two parts, Experimental Details and Supplementary Figures and Tables. Figure S1. Cluster results for DBSCAN & Illustration of the scheme named up, middle, and down. Figure S2. The values of total energy, temperature, pressure, and density from trajectory file of the last 25 ns MD simulation for the *G. sulfurreducens* pilin. Figure S3. Spatial distribution of aromatics and charged residues. Orange and yellow spheres represent the aromatic residues distributed in the electron transfer pathway and the aromatic residues not distributed in the electron transfer pathway, respectively. Green sticks represent the charged residues. Charged amino acids (E5, R28, 30K, 39D, R41, 44K, E48, and 53D) in close proximity to the aromatic amino acids of the charge transport path. Figure S4. The frontier molecular orbitals (HOMO-1, HOMO and LUMO, LUMO+1) in aromatic residue dimers (middle part of GS1) by DFT calculations at the level of CAM-B3LYP/6-31g. Table S1. Calculated microscopic electronic structure parameters for interresidue hole/electron transfer by the DFT method with CAM-B3LYP functional and different basis sets.
