# Supplementary material for: Direct Extracellular Electron Transfer of the Geobacter sulfurreducens Pili Relevant to Interaromatic Distances
Source: Biomed Res Int. 2019 Nov 11;2019:6151587. doi: 10.1155/2019/6151587 (PMC6925702; doi:10.1155/2019/6151587)
Supplement: Supplementary Materials — The supplementary material file consists of two parts, Experimental Details and Supplementary Figures and Tables. Figure S1. Cluster results for DBSCAN & Illustration of the scheme named up, middle, and down. Figure S2. The values of total energy, temperature, pressure, and density from trajectory file of the last 25 ns MD simulation for the G. sulfurreducens pilin. Figure S3. Spatial distribution of aromatics and charged residues. Orange and yellow spheres represent the aromatic residues distributed in the electron transfer pathway and the aromatic residues not distributed in the electron transfer pathway, respectively. Green sticks represent the charged residues. Charged amino acids (E5, R28, 30K, 39D, R41, 44K, E48, and 53D) in close proximity to the aromatic amino acids of the charge transport path. Figure S4. The frontier molecular orbitals (HOMO-1, HOMO and LUMO, LUMO+1) in aromatic residue dimers (middle part of GS1) by DFT calculations at the level of CAM-B3LYP/6-31g. Table S1. Calculated microscopic electronic structure parameters for interresidue hole/electron transfer by the DFT method with CAM-B3LYP functional and different basis sets. [file 6151587.f1.docx]

Supporting information

**Direct extracellular electron transfer of the *Geobacter sulfurreducens* pili relevant to inter-aromatic distances**

*Chuanjun Shu^a,c,1^,Qiang Zhu ^b,1^, Ke Xiao^a^, Yue Hou^a^, Haibo Ma^b,*^, Jing Ma^b^, and Xiao Sun^a,*^*

*^a^State Key Laboratory of Bioelectronics, School of Biological Science and Medical Engineering, Southeast University, Nanjing, 210096, P. R. China.*

*^b^Institute of Theoretical and Computational Chemistry, School of Chemistry and Chemical Engineering and Key Laboratory of Mesoscopic Chemistry of MOE, Nanjing University, Nanjing 210023, P. R. China.*

*^c^Department of Bioinformatics, School of Biomedical Engineering and Informatics, Nanjing Medical University, Nanjing, 211166, China*

*^1^These authors contributed equally to this work.*

*** Correspondence:** *Xiao Sun, Email: xsun@seu.edu.cn;* *Haibo Ma, Email: haibo@nju.edu.cn.*

# 1. Experimental details

The symmetric definition files could be extracted from the GC pilus structure (PDB ID: 2HIL) by running the make_symmdef_file.pl script:

>perl $rosettadir/rosetta_source/src/apps/public/symmetry/make_symmdef_file.pl -m HELIX -p 2HIL.pdb -r 20.0 -a A -b B -t 7 > sym.def

Step 1: Low-resolution phase

Execution of the Rosetta procedure:

>$rosettadir/main/source/bin/SymDock.linuxgccrelease \

-in:file:s subunit.pdb \

-database $rosettadir/main/database \

-symmetry:symmetry_definition symm.def \

-packing:ex1 \

-packing:ex2aro \

-out:nstruct 3000 \

-out:pdb \

-symmetry:initialize_rigid_body_dofs \

-constraints:cst_file lowres.cst \

-constraints:cst_weight 10.0 \

-docking:dock_lowres_filter 1.0 1.0 1.0 \

-mute core

Step 2: High-resolution phase

Execution of the Rosetta procedure

>$rosettadir/main/source/bin/SymDock.linuxgccrelease \

-in:file:s starting_INPUT.pdb \

-database $rosettadir/main/database \

-symmetry:symmetry_definition symm_highres.def \

-packing:ex1 \

-packing:ex2aro \

-out:nstruct 1000 \

-out:file:fullatom \

-symmetry:initialize_rigid_body_dofs \

-constraints:cst_file lowres.cst \

-constraints:cst_weight 10.0 \

-constraints:cst_fa_file highres.cst \

-constraints:cst_fa_weight 100.0 \

-docking:dock_lowres_filter 1.0 1.0 1.0 \

-mute core \

-docking:kick_relax \

-docking:dock_ppk

# 2. Figures and tables

| 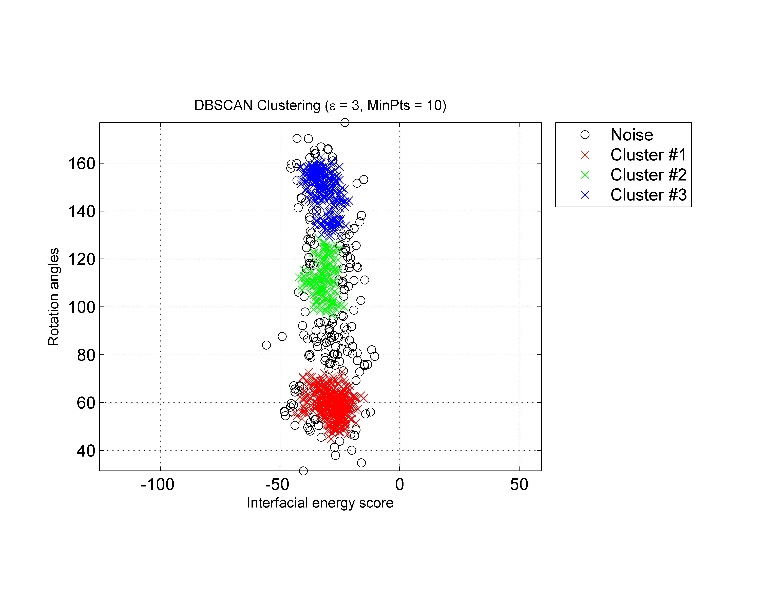 | 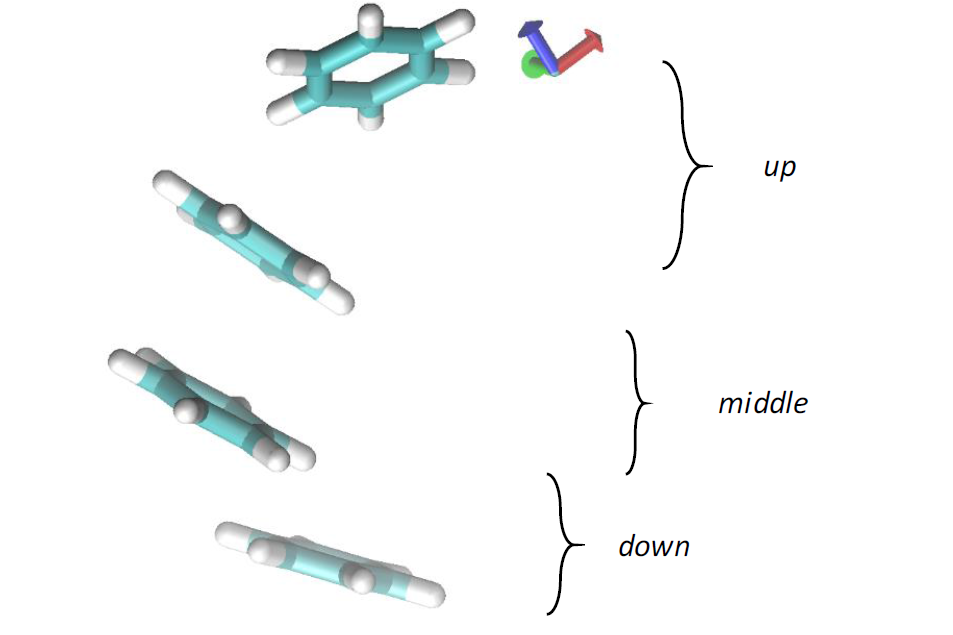 |
| --- | --- |

Figure S1. Cluster results for DBSCAN & Illustration of the scheme named up, middle and down.


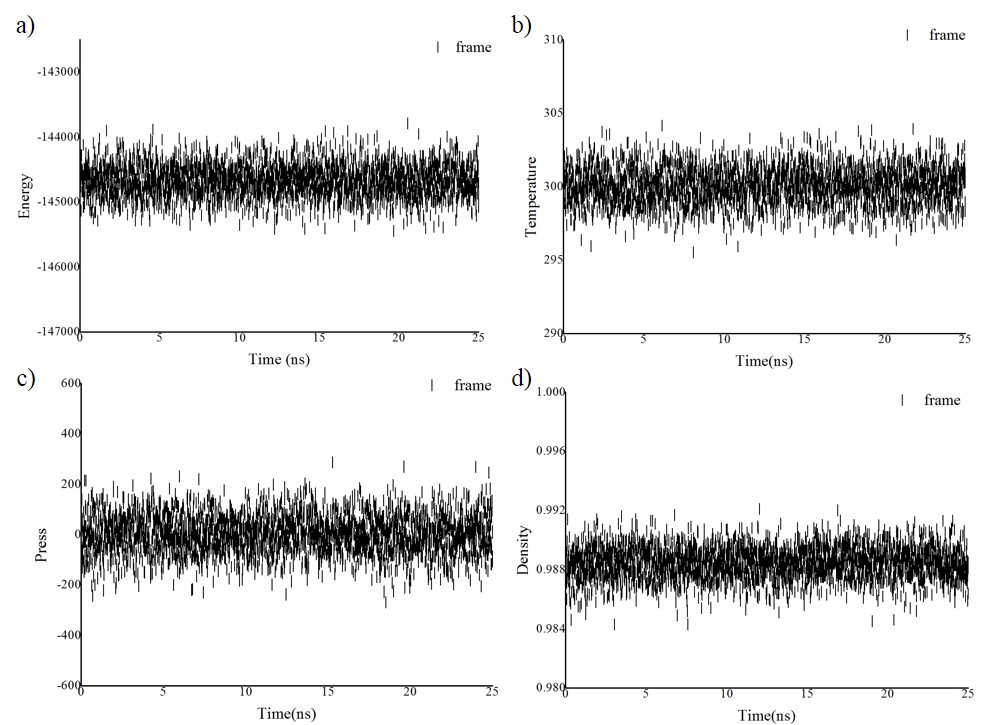


Figure S2. The values of total energy, temperature, pressure, and density from trajectory file of the last 25 ns MD simulation for the G. *sulfurreducens* pilin.


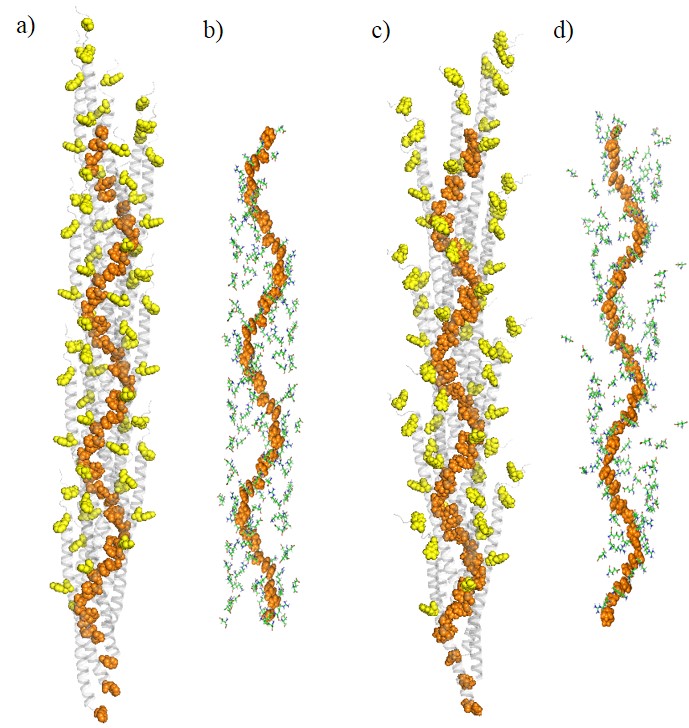


Figure S3. Spatial distribution of aromatics and charged residues. Orange and yellow spheres represent the aromatic residues distributed in the electron transfer pathway and the aromatic residues not distributed in the electron transfer pathway, respectively. Green sticks represent the charged residues. Charged amino acids (E5, R28, 30K, 39D, R41, 44K, E48, and 53D) in close proximity to the aromatic amino acids of the charge transport path.

Figure S4. The frontier molecular orbitals (HOMO-1, HOMO and LUMO, LUMO+1) in aromatic residue dimers (middle part of GS1) by DFT calculations at the level of CAM-B3LYP/6-31g.

Table S1. Calculated microscopic electronic structure parameters for inter-residue hole/electron transfer by the DFT method with CAM-B3LYP functional and different basis sets.

|  |  | hole | | electron | |
| --- | --- | --- | --- | --- | --- |
|  |  | 6-31g | 6-311++g(d, p) | 6-31g | 6-311++g(d, p) |
| t/10^-2^ eV | up (GS1) | 1.56 | 2.04 | 0.33 | 1.44 |
|  | middle (GS1) | 6.32 | 6.20 | 0.09 | 1.64 |
|  | down (GS1) | 2.99 | 3.43 | 0.27 | 1.23 |
|  | up (GS2) | 1.46 | 2.03 | 0.29 | 1.40 |
|  | middle (GS2) | 4.27 | 4.16 | 0.15 | 1.65 |
|  | down (GS2) | 3.63 | 4.04 | 0.34 | 1.24 |
| λ/ eV | up (GS1) | 0.16 | 0.16 | 20.70×10^-2^ | 0.08×10^-2^ |
|  | middle (GS1) | 0.16 | 0.16 | 20.69×10^-2^ | 0.07×10^-2^ |
|  | down (GS1) | 0.17 | 0.16 | 20.29×10^-2^ | 0.04×10^-2^ |
|  | up (GS2) | 0.16 | 0.16 | 20.65×10^-2^ | 0.07×10^-2^ |
|  | middle (GS2) | 0.17 | 0.16 | 20.65×10^-2^ | 0.08×10^-2^ |
|  | down (GS2) | 0.17 | 0.16 | 20.70×10^-2^ | 0.04×10^-2^ |

By using different Pople basis set without (6-31g) and with (6-311++g(d, p)) diffuse and polarization functions, we found that both of them can present close results for hole transfer parameters. The large difference for electron transfer parameters by 6-31g and 6-311++g(d, p) can be ascribed to the fact that the large basis set with many diffuse functions creates many spurious virtual molecule orbitals and the consequent many spurious excitations or ionizations, which has been widely noticed by recent computations^1^.

**References:**

1. D. J. F. Walker, R. Y. Adhikari, D. E. Holmes, J. E. Ward, T. L. Woodard, K. P. Nevin and D. R. Lovley, *ISME J*, 2017, **2017**, 141.
